# Comparative miRNA Analysis of Urine Extracellular Vesicles Isolated through Five Different Methods

**DOI:** 10.3390/cancers8120112

**Published:** 2016-12-10

**Authors:** Felix Royo, Izzuddin Diwan, Michael R. Tackett, Patricia Zuñiga, Pilar Sanchez-Mosquera, Ana Loizaga-Iriarte, Aitziber Ugalde-Olano, Isabel Lacasa, Amparo Perez, Miguel Unda, Arkaitz Carracedo, Juan M. Falcon-Perez

**Affiliations:** 1 Exosomes Lab, CIC bioGUNE, CIBERehd, Derio 48160, Spain; 2 Abcam, One Kendall Square, Cambridge, MA 02139, USA; Izzuddin.Diwan@abcam.com (I.D.); Michael.Tackett@abcam.com (M.R.T.); 3 Cancer Cell Signaling and Metabolism Lab, CIC bioGUNE, Derio 48160, Spain; pzuniga@cicbiogune.es (P.Z.); psanchez@cicbiogune.es (P.S.-M.); acarracedo@cicbiogune.es (A.C.); 4 Urology Service, Basurto University Hospital, Bilbao 48013, Spain; ana.loizagairiarte@osakidetza.net (A.L.-I.); aitziber.ugaldeolano@osakidetza.net (A.U.-O.); ISABEL.LACASAVISCASILLAS@osakidetza.net (I.L.); amparo.perezfernandez@osakidetza.net (A.P.); jesusmiguel.undaurzaiz@osakidetza.net (M.U.); 5 IKERBASQUE Basque Foundation for Science, Bilbao 48013, Spain

**Keywords:** extracellular vesicles, exosomes, urine, miRNA, isolation methods

## Abstract

Urine extracellular vesicles are a valuable low-invasive source of information, especially for the cells of the genitourinary tract. In the search for biomarkers, different techniques have been developed to isolate and characterize the cargo of these vesicles. In the present work, we compare five of these different isolation methods (three commercial isolation kits, ultracentrifugation, and lectin-based purification) and perform miRNA profiling using a multiplex miRNA assay. The results showed high correlation through all isolation techniques, and 48 out of 68 miRNAs were detected above the detection limit at least 10 times. The results obtained by multiplex assay were validated through Taqman qPCR. In addition, using this technique combined with a clinically friendly extracellular vesicle (uEV)-enrichment method, we performed the analysis of selected miRNAs in urine from patients affected with bladder cancer, benign prostate hyperplasia, or prostate cancer. Importantly, we found that those miRNAs could be detected in almost 100% of the samples, and no significant differences were observed between groups. Our results support the feasibility of analyzing exosomes-associated miRNAs using a methodology that requires a small volume of urine and is compatible with a clinical environment and high-throughput analysis.

## 1. Introduction

Urine has very interesting features as a source of biomarkers; it is easy to collect, both protein and metabolites are present in it, and it also contains extracellular vesicles (uEVs) [1,2]. These come mostly from renal and urethral cells [3], but also may filter directly from systemic circulation [4]. Therefore, unlike tissue biopsy, uEVs provide a full representation of the entire urinary system and can be used to provide markers for urinary tract conditions [3,5,6,7] as well as systemic conditions [4,8]. In the search for RNA biomarkers in urine, the study of uEVs is of particular importance, since they provide protection against degradation [9] in an environment with high RNAse [10] content.

One of the major difficulties in the application of uEV research into the clinical environment is the variety and technical complexity of some of the proposed methods of uEV isolation [11]. However, a growing number of commercial solutions have appeared, adding a new source of variability, since the results achieved with these techniques can differ from traditional isolation techniques [11,12].

To address this question, we previously studied the variability between five different methods employed to isolate uEVs [13]—three methods using commercial kits: Urine Exosome RNA Isolation Kit (NORGEN, Biotek Corp., Thorold, ON, Canada, cited as NOR), Total Exosome Isolation Solution (ThermoFisher, Waltham, MA, USA, cited as INV), Exoquick-TC (System Biosciences, Palo Alto, CA, USA, cited as EXQ); ultracentrifugation (cited as CEN); and a lectin-based purification, exploiting affinity for glycosylated proteins enriched on the surface of uEVs [14,15] (cited as LEC). In that previous work, we compared the recovery of uEV protein markers and messenger RNAs, using each of the methods to analyze samples obtained from healthy donors. Our results showed that different methods had different affinity for protein markers, and regarding messenger RNA, the purification using NOR was able to detect more genes in more samples than the others.

In the present work, using the same set of samples, we studied the miRNA recovery from each technique. We employed the Multiplex Circulating miRNA assay (Abcam PLC, Cambridge, UK), and we validated the results using Taqman qPCR. The Multiplex Circulating miRNA assay enables the simultaneous profiling of up to 68 miRNAs directly from small volumes (10–40 μL) of biofluid or enriched uEVs, thus eliminating the need for—and introduced bias of—RNA isolation. Furthermore, the assay is high-throughput, enabling the easy simultaneous profiling of dozens of miRNAs in hundreds of samples. In addition, combining one of the methodologies with qPCR, a group of selected miRNAs previously related to cancer were evaluated in urine samples from patients with bladder cancer (BCa) prostate cancer (PCa), or benign prostatic hyperplasia (BPH).

## 2. Results

The protein and messenger RNA characterization of the uEVs isolated from each method was described previously [13]. In this previous work, major differences were found in the exosomal markers enriched by each method, and remarkably, messenger RNA was more abundant in the uEVs preparation enriched by using the NOR method. In the current work, we focus on the miRNA content of the uEVs obtained by the different methods.

### 2.1. Multiplex miRNA Assay

Firstly, we attempted to profile miRNAs directly from the urine. Interestingly, as shown in Figure S1, while some targets were detected directly from the urine samples without any enrichment, we found that a much more robust profile of miRNAs could be determined by purifying (and enriching for) uEVs.

**Figure 1:**
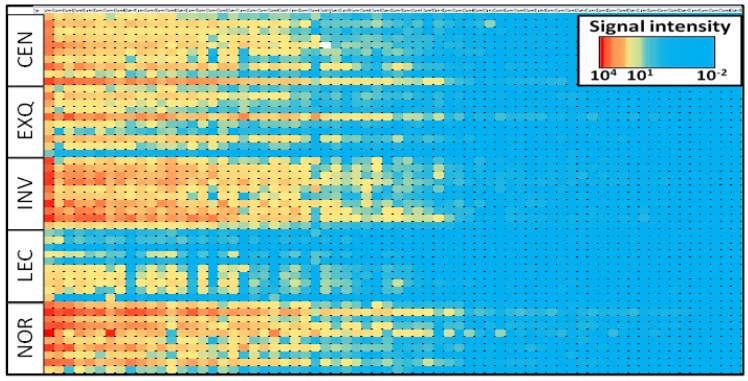
Heatmap analysis of multiplex miRNA assay. The color code corresponds to the signal intensity obtained in the assay, and samples were grouped by isolation method. Each square corresponds to a single miRNA and sample. CEN: ultracentrifugation; EXQ: Exoquick-TC; INV: Total Exosome Isolation Solution; LEC: lectin-based purification; NOR: Urine Exosome RNA Isolation Kit.

After showing the benefits of uEV enrichment, we analyzed the miRNA profiling of uEVs isolated by different existing methods. Five different uEV purification methods were used on each of ten independent urine samples, and a total of 68 miRNAs were profiled. The heatmap in Figure 1 presents the signal intensity achieved for each sample and miRNA, grouped by isolation method. Up to 48 miRNAs from the 68 miRNAs assayed were detected at least 10 times above the detection limit of the multiplex assay (Table S1). The heatmap shows that the LEC method yielded the lowest signal intensity for most of the assayed miRNAs, while the other methods demonstrated comparable performance to each other.

**Figure 2:**
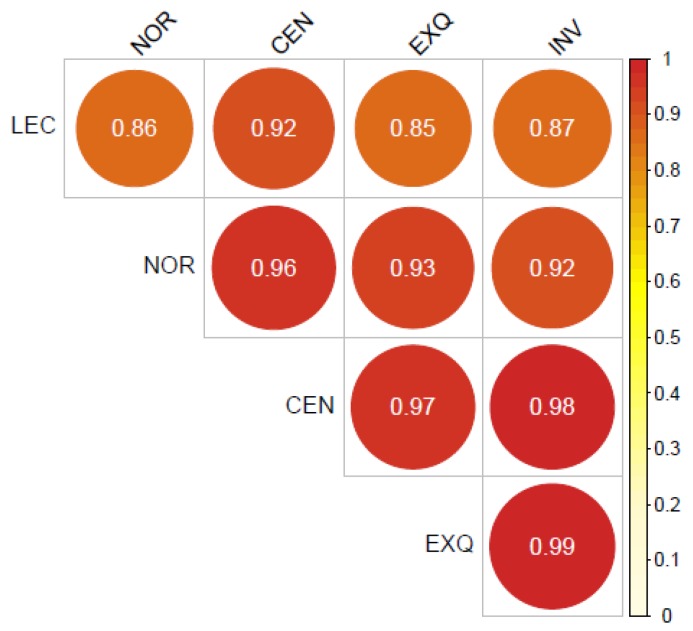
Correlation matrix between different methods. The r coefficients show that LEC purification performed slightly different than the other methods. All the correlations are highly significant (*p* < 0.001).

We then performed a correlation analysis between the values obtained for each detected miRNA by each isolation method. The r coefficients were above of 0.9 between all the methods, except for LEC, which presented slightly lower values for most of the comparisons (Figure 2). For that reason, we decided to remove this group from ANOVA comparisons, and explore if there were differences between the other four methods for specific miRNAs.

**Figure 3:**
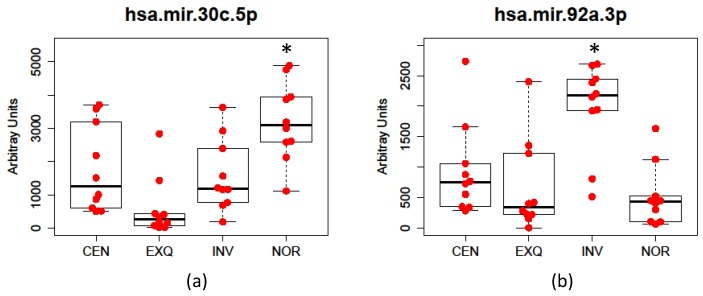
ANOVA analysis of the performance of the different methods for (**a**) hsa-mir-30c-5p and (**b**) hsa-mir-92a-3p detection. Note: in panel (**a**), NOR is significantly different from the other treatments, while in panel (**b**) INV is different from the rest of the treatments (*p* < 0.05, by post-hoc comparisons using Bonferroni correction).

The results showed that only two of the tested miRNAs had statistically significant differences between the isolation methodologies: hsa-mir-30c-5p, with an adjusted *p*-value of 0.007, and hsa-mir-92a-3p, with an adjusted *p*-value of 0.010. Thus, NOR detected hsa-mir-30c-5p better than the other methods, while hsa-mir-92a-3p was more enriched using the INV method (Figure 3). Comparing intensity values obtained in the multiplex miRNA assay with the presence of proteins in each sample (observed by Western blot and quantified by densitometry in [13]), we could not observe a correlation between miRNA abundance and the presence of contaminant Tamm–Horsfall protein (THP). However, we observed a certain degree of correlation between miRNA abundance and certain membrane markers, but with differences depending on the isolation method. For instance, we observed the highest correlations between miRNAs abundance and AIP1 (Alix) for samples isolated by CEN method, AIP1, AQP2, and CD9 for EXQ, AIP1 and CD63 for LEC, CD26 and CD63 for INV, and CD26 and TSG101 for NOR (Figure S2). These data suggest both the association between miRNA and vesicles, as well as the different affinity of different methods for certain EV populations that we also observed in our previous work [13].

### 2.2. Validation Using Taqman qPCR

In order to validate the results obtained using the Multiplex Circulating miRNA assay, we planned a validation by a different technology based on Taqman qPCR, employing a subset of three samples with the same RNAs assayed in the multiplex assay. We selected nine miRNAs, including those that behave differentially between assays, some of the miRNAs that provided more intense signals (see Table S1), and some miRNAs with medium or low signal intensity—including hsa-mir-122-5p, which was detected in few samples.

**Figure 4:**
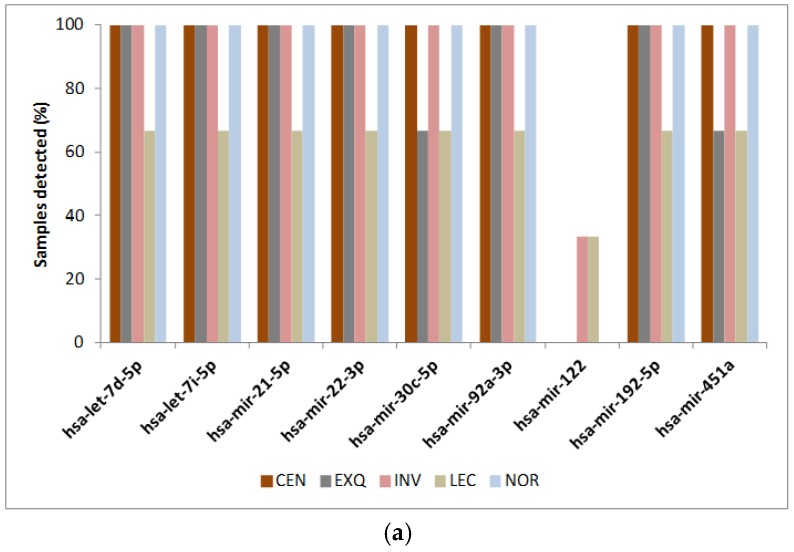

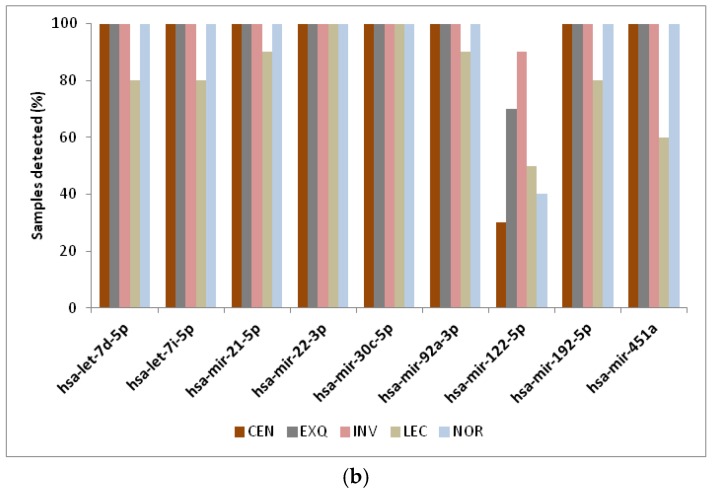
Percentage of detection for each miRNA, employing (**a**) Taqman qPCR or (**b**) Multiplex Circulating miRNA Assay (Abcam).

**Figure 5:**
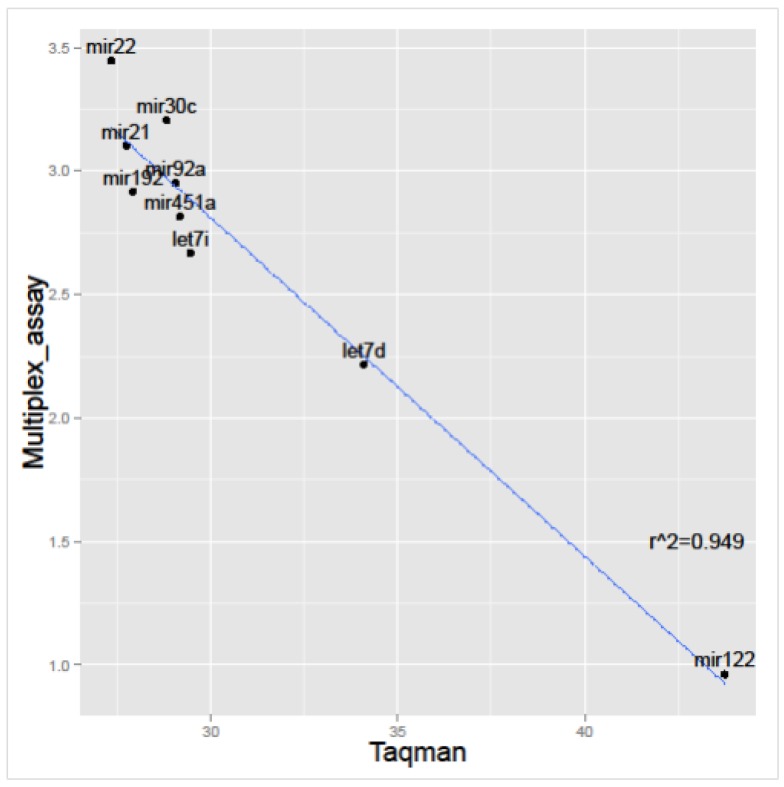
Correlation between signal intensity (Log_10_ transformed) achieved by multiplex miRNA assay and *C*_t_ values obtained by Taqman qPCR, using the same RNAs.

The results indicated that the detection of each miRNA was similar using both techniques, although multiplex assay detected miRNAs in a slightly greater number of samples (Figure 4). More importantly, the correlation between both techniques—comparing signal intensity in the multiplex assay with the average *C*_t_ number detected for each miRNA—presented a very high correlation, indicating that both techniques achieve similar values (Figure 5). We present this correlation by each isolation method in Figure S3. It is possible to observe that all of the isolation methods have a high correlation coefficient between signal intensity in the Multiplex miRNA assay and Taqman qPCR (*C*_t_ values) with the exception of LEC, the method that recovers fewer miRNAs.

### 2.3. RNAse Protection Assay

We evaluated the presence of miRNAs inside uEVs by treating healthy urine samples with RNAse in the presence of proteinase K or the detergent Triton X-100 (see Section 4 for details). Most amplified miRNAs were resistant to treatment with Proteinase K plus RNAse. However, the amplification of miRNAs was abrogated when RNAse treatment was performed in the presence of a detergent (Figure S4), indicating that they were protected in the uEVs. Of note, we also observed a clear reduction for let-7d-5p with the RNAse plus proteinase K treatment, although there is also some degree of protection—a protection that is lost when the sample is treated with Triton X-100, suggesting that miRNA was also present in vesicles.

### 2.4. Presence of miRNAs in Patient Samples

Since the results of the multiplex miRNA assay allowed us to select a certain number of miRNAs present in most urine samples, and some of those miRNAs have been previously related to malignancies in the genitourinary tract (Table S2), we decided to test whether we could use Taqman qPCR to detect those miRNAs in samples from patients with different genitourinary tract conditions. In total, we analyzed samples from eight patients classified as “non-tumoral” (NT), six as benign prostate hyperplasia (BPH), seven patients presented malignant growth in the urinary bladder (BCa), and seven patients presented prostate tumor (PCa). Moreover, we again employed 10 mL of voided urine and the fastest extraction technique, which was the NOR method. Combining those techniques, we detected most of the miRNAs in almost all the samples, as detailed in Figure S5.

**Figure 6:**
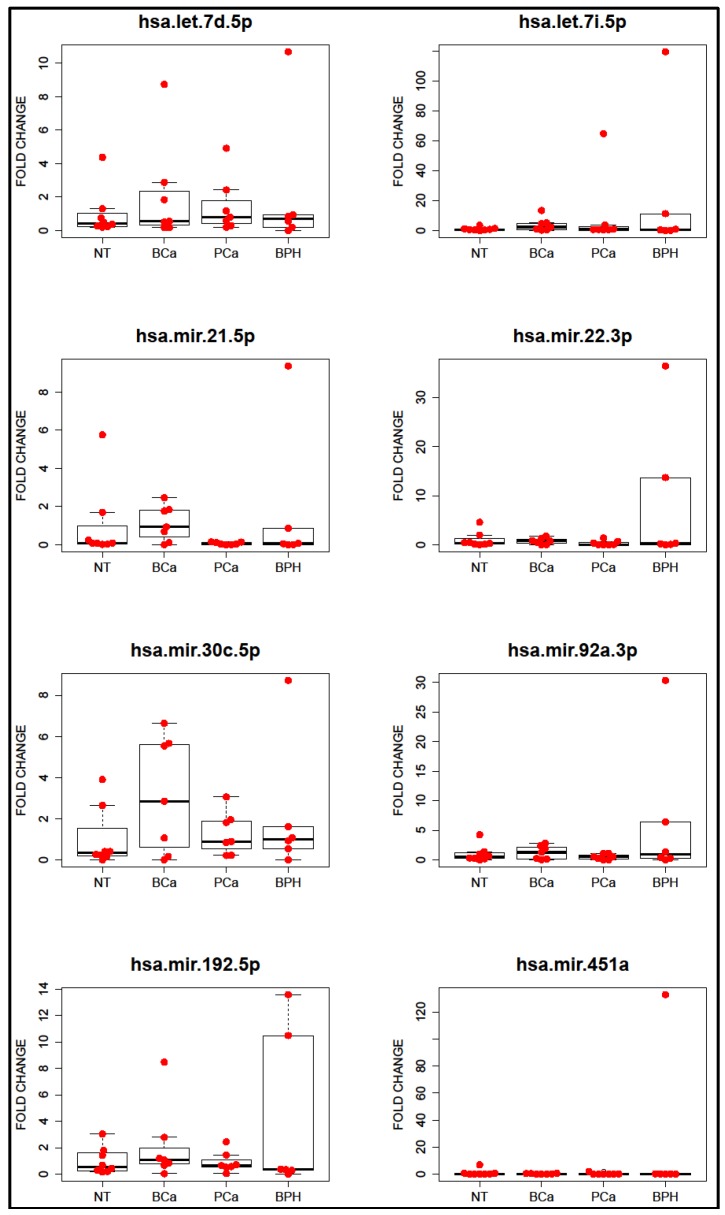
miRNAs analysis of extracellular vesicles (uEVs)-enriched preparations obtained of different genitourinary track pathologies. NT (non-tumoral, patients without malignancies), BCa (bladder cancer), PCa (prostate cancer), and BPH (benign prostate hyperplasia). Fold changes were calculated using cel-mir-39c-3p as housekeeping, and the data was normalized against the average of the NT group, so the average of the group is 1 for each miRNA. ANOVA did not find any significant differences amongst groups.

The comparison between types of patients did not yield any significant result. Unfortunately, the large individual variation and the low number of samples made it impossible to achieve any conclusion about the possible role of the selected miRNA as markers of disease. We could highlight the presence of two samples from patients with BPH with more miRNAs than the rest of samples, as well as the increase of hsa-mir-30c-5p in patients with bladder cancer (Figure 6).

## 3. Discussion

The present work focused on the miRNA content enriched by different methods employed for the purification of uEVs. In a previous work, we observed that each method had a greater affinity for uEVs harboring certain membrane markers, even though most markers were present in all preparations, and individual heterogeneity was observed. As a summary of our previous work, we found that NOR had a unique affinity for AIP1 (Alix), CEN for CD63, LEC for CD9, and INV was somehow balanced between markers more enriched by NOR and markers more enriched by CEN [13].

Regarding messenger RNA, we could amplify a set of genes with different methods; meanwhile, other subsets of genes were only amplified by NOR, making this method advisable to purify messenger RNA from urine for biomarker screening [13]. However, by analyzing the miRNA content of uEVs in the present work, the differences between methods are less obvious, and only LEC is clearly less efficient than the other tested methods. This suggests that the enrichment of CD9 negatively correlates with the presence of miRNAs. Regarding the presence of miRNAs, the Multiplex miRNA assay from Abcam detected 48 (out of 68) miRNAs clearly above the detection limit of the assay. Among the tested miRNAs, we found some already described as abundant in the exosomal fraction, and consistently appeared in our assay as the most enriched miRNAs by different methods; these included hsa-miR-30a-5p, hsa-miR-30c-5p, hsa-miR-192-5p, and hsa-miR-451a (Table S1) [16]. Interestingly, hsa-miR-30c-5p—which was enriched by NOR in our analysis—was also found in Cheng et al.’s study using NOR. They also described hsa-miR-192-5p and hsa-mir-451 as some of the more abundant in the fraction captured by NOR. However, hsa-mir-451a is also highly present in the free-cell fraction, implying the capability of NOR to isolate not only miRNA associated to vesicles, but also free miRNA [16]. This may explain our previous observation of certain messenger RNAs that were only detected by NOR [13]. In any case, it is the isolation from 10 mL of urine that makes the detection possible, so enrichment is necessary to perform the analysis—a conclusion in agreement with their previous observation [16]. According to our results, a number of miRNAs can be detected repeatedly from 10 mL of previously frozen urine using commercial kits that do not require an ultracentrifuge, which may be an important point when the goal is to apply the findings in a clinical environment [11]. Importantly, a quick characterization of a panel of miRNAs can be done by the Multiplex Circulating miRNA assay of Abcam, since the results are also consistent with Taqman qPCR results. Therefore, the results could be validated in a large cohort of samples by Taqman qPCR, a technique that may be more appropriate for assessing a small number of miRNAs.

In the present work, we failed to find any differences between samples from different types of patients. Certainly, the number of samples and the low number of miRNAs tested is a clear limitation of this study. However, one of the most interesting insights from our result is the observation of some samples from BPH patients with higher amounts of miRNAs, a phenomenon consistent with our previous study, where we also found BPH samples with a greater amount of mRNAs [7]. On the other hand, the miRNA hsa-miR-30c-5p has been described as downregulated in the tissue of the tumor when compared with the surrounding tissue of the bladder [17]—a phenomenon that may explain its enrichment in uEVs from patients with BCa, even if those changes do not reach statistical significance. Similarly, hsa-miR-21-5p was also slightly higher in our BCa samples, and it has also been previously observed to be enriched in bladder cancer uEVs [18].

To conclude, we remark that the importance of our results is the ability to characterize the miRNA content in previously frozen urine in such small volumes as 10 mL by methods that do not require ultracentrifugation, in a reproducible manner and compatible with high-throughput analysis.

## 4. Materials and Methods

### 4.1. Human Urine Samples

Samples were taken from ten healthy volunteers, obtained from the first urine of the morning, corresponding to our previous work [13]. For the present study, we also collected samples from 28 patients cited for exploratory prostate biopsy or urinary endoscopy. All samples were obtained by spontaneous micturition. The study was approved by the Basque Ethical Committee for Clinical Research (CEIC code 11–12 and 14–14). All urine samples were centrifuged at 2000 × *g* for 10 min, filtered through a 0.22-μm pore membrane, and immediately frozen at −80 °C. Samples from eight patients were classified as “non-tumoral” (NT), six as benign prostate hyperplasia (BPH), seven patients presented malignant growth in the urinary bladder (BCa), and seven patients presented prostate tumor (PCa). The diagnosis was based on the results of histological examination performed by a pathologist at the urology department of Basurto University Hospital.

### 4.2. uEV Enrichment

The purification of uEVs from urine samples by different methods was performed in a previous work [13]. Briefly, to compare different uEV enrichment methods, urine samples from ten healthy donors were collected and analyzed independently. A 100 mL urine sample from each donor was thawed and vortexed. Afterwards, each sample was split into 10 aliquots of 10 mL each to perform the five protocols (each in duplicate). Ultracentrifugation was carried out in a single step, using a Beckman–Coulter 70Ti rotor. The extraction with ExoQuick-TC Exosome Precipitation Solution (System Biosciences) was performed per manufacturer’s instructions, as was the extraction with Total Exosome Isolation solution (ThermoFisher Scientific, Waltham, MA, USA). Certain modifications were introduced to the protocol for Urine Exosome RNA Isolation Kit (NORGEN, Biotek Corp.). Once urine was mixed with the slurry NORGEN component, we divided each 10 mL aliquot into three aliquots. For the extraction of RNA, we used 7 mL of the mix, and the final volume used for elution was 35 μL. The last isolation method involved the extraction with biotinylated *Solanum tuberosum* (potato) lectin (STL) (Vector Laboratories, Burlingame, CA, USA), and it was performed as described previously [16]. Each method was assigned an abbreviation: CEN for ultracentrifugation, NOR for NORGEN, INV for Total Exosome Isolation Solution, EXQ for Exoquick-TC, and LEC for STL purification. For the samples obtained from patients, we performed RNA extraction directly as described in the instructions of the Urine Exosome RNA Isolation Kit (NORGEN, Biotek Corp.).

### 4.3. RNA Isolation

The NORGEN-based procedure was performed as described in the previous section. During the remaining four procedures, after the last centrifugation or magnetic bead recovery, each sample was suspended in 100 μL of exosome resuspension buffer (ERB) from the Total Exosome RNA and Protein Isolation Kit (ThermoFischer Scientific). A 70-μL aliquot of the suspension was used for RNA extraction, per the manufacturer’s protocol, with a final elution volume of 35 μL.

### 4.4. Multiplex miRNA Assay

A panel of 68 miRNAs (listed in Table S1) was used for profiling 40 μL of urine or 15 μL of RNA samples of uEVs isolated by the different methods. For each sample run, Firefly Particles (35 μL) were added to a well of a 96-well filter plate and filtered. Next, 25 μL of Hybe Buffer and 25 μL of sample was added to each well. The plate was incubated at 37 °C for 60 min with shaking. After rinsing, 75 μL of 1× Labeling Buffer was added to each well. The plate was incubated at room temperature for 60 min with shaking. After additional rinses, 65 μL of 95 °C RNAse-free water was added twice to each well to elute the ligated sample. Thirty microliters of this meltoff was mixed with 20 μL PCR master mix. The mixture underwent 27 cycles of PCR amplification followed by six cycles of asymmetric amplification. Next, 60 μL of Hybe Buffer was added back to each well of the original particles, followed by 20 μL of the PCR product, and the plate was incubated at 37 °C for 30 min with shaking. After rinsing, 75 μL of 1× Reporting Buffer was added to each well, and the plate incubated at room temperature for 15 min with shaking. After final rinses, 175 μL of Run Buffer was added to each well. Particles were scanned on an EMD Millipore Guava 8HT flow cytometer. Raw output was background subtracted and subsequently employed in statistical calculations.

### 4.5. Taqman miRNA Assay

Reverse transcription of the miRNA was performed using TaqMan Advanced miRNA cDNA Synthesis Kit (ThermoFisher Scientific), following the manufacturer’s recommendations. We employed 2 μL of RNA for each reaction, and we added 1 μL of 0.1 nM cel-miR39-3p spiked in the mix that we used as a control to calculate fold changes. The TaqMan reactions were performed using the Taqman Advance miRNA assays (ThermoFisher Scientific) for the following miRNAs (hsa-let-7d-5p, hsa-let-7i-5p, hsa-mir-21-5p, hsa-mir-22-3p, hsa-mir-30c-5p, hsa-mir-92a-3p, hsa-mir-122-5p, hsa-mir-192-5p, hsa-mir-451a), and qPCR was performed in a Via7 using TaqMan^®^ Fast Advanced Master Mix (ThermoFisher Scientific). *C*_t_ values of duplicates were averaged and used in further calculations.

### 4.6. RNAse Protection Assay

To evaluate the presence of miRNA inside the extracellular vesicles, we compared the miRNA amplification from urine treated with proteinase K and RNase with untreated urine, with a protocol adapted from [19]. We also included an additional sample treated with RNAse and 0.1% Triton X-100, a treatment that degrades RNA inside vesicles [20]. Briefly, 30 mL of urine from a healthy donor was divided into three aliquots of 10 mL. One of the aliquots was treated with 0.05 mg/mL of Proteinase K at 37 °C for 10 min, then the reaction was stopped by adding 5 mM of phenylmethylsulfonyl fluoride (PMSF) and heat inactivation at 90 °C for 5 min. Another aliquot was treated with 0.1% Triton X-100. To the third aliquot, only PMSF was added and used as control. Then, 0.1 mg/mL of RNAse A was added to the samples containing Proteinase K or Triton X-100. The three samples (Proteinase K, Triton X-100, and Control) were incubated at 37 °C for 20 min. Afterward, RNA was extracted using NOR method, and cDNA and Taqman qPCR were performed as previously described.

### 4.7. Statistical Analysis

Correlation matrices and associated *p*-values were obtained using the cor function and plotted using the corrplot package of R v3.1.0 software (2014-04-10, R Foundation for Statistical Computing, Vienna, Austria). For correlation, *p*-values lower than 0.05 were considered significant. ANOVA was performed using the aov function from the basic packages of R v3.1.0 software.

## 5. Conclusions

We have shown that four methods currently available for the enrichment of EVs have been equally successful in obtaining a miRNA profile from 10 mL of frozen-stored urine. Importantly, miRNA profiling was only possible after EV isolation, and not directly from urine. The assay employed—a commercial Multiplex assay from Abcam—was also validated by Taqman, pointing that this platform offers reliable results, and therefore it can be used for an extensive miRNA profiling in a good number of samples simultaneously. Finally, the miRNAs found in healthy donors were also found in patients of bladder and prostate cancers, providing an easy pipeline for the miRNA analysis of urinary EVs suitable for clinical environments.

## Supplementary Materials

The following are available online at http://www.mdpi.com/2072-6694/8/12/112/s1: Figure S1: Differences in miRNA detection between urine, and RNA isolated from EVs of the same urine sample; Figure S2: Matrix correlation between miRNA and protein markers abundance; Figure S3: Correlation between Multiplex miRNA assay and Taqman qPCR for each isolation method; Figure S4: RNase protection assay for urine samples; Figure S5: Percentage of positive samples where each miRNA was detected; Table S1: List of miRNA assayed in the Multiplex miRNA assay of Abcam; Table S2: References supporting association between selected miRNAs and cancer.
