# Supplementary material for: Comparative miRNA Analysis of Urine Extracellular Vesicles Isolated through Five Different Methods
Source: Cancers (Basel). 2016 Dec 10;8(12):112. doi: 10.3390/cancers8120112 (PMC5187510; doi:10.3390/cancers8120112)
Supplement: Supplementary file 1 [file cancers-08-00112-s001.pdf]

Supplementary Materials: Comparative miRNA Analysis of Urine Extracellular Vesicles Isolated through Five Different Methods

Felix Royo, Izzuddin Diwan, Michael R Tackett, Patricia Zuñiga, Pilar Sanchez-Mosquera, Ana Loizaga-Iriarte, Aitziber Ugalde-Olano, Isabel Lacasa, Amparo Perez, Miguel Unda, Arkaitz Carracedo and Juan M Falcon-Perez

Table S1. Supplemental Table S1. miRNAs assayed by Multiplex miRNA assay of Firefly, and signal intensity for each isolation method (Mean +/− SEM). Last column indicates detection limit of detection for each miRNA. CEN: ultracentrifugation; EXQ: Exoquick-TC; INV: Total Exosome Isolation Solution; LEC: lectin-based purification; NOR: Urine Exosome RNA Isolation Kit.

TYPE	CEN	EXQ	INV	LEC	NOR	AVERAGE	LIMIT
hsa-mir-22-3p	3070.82 ± 158.93	1818.82 ± 156.96	4467.62 ± 199.4	515.41 ± 223.51	3943.43 ± 215.65	2763.22	2.48
hsa-mir-30c-5p	1768.45 ± 26.28	593.3 ± 26.01	1567.35 ± 32.12	523.77 ± 39.47	3207.26 ± 38.74	1532.03	0.61
hsa-mir-16-5p	1486.8 ± 42.34	1051.28 ± 51.95	2265.55 ± 57.34	210.53 ± 57.21	2585.24 ± 56.43	1519.88	9.07
hsa-mir-29c-3p	1655.62 ± 294.34	892.9 ± 292.4	2322.51 ± 300.42	355.7 ± 374.25	1870.85 ± 439.05	1419.51	0.87
hsa-mir-29a-3p	1374.69 ± 106.3	786.96 ± 106.29	2059.19 ± 168.19	245.08 ± 241.5	1827.04 ± 234.07	1258.59	0.8
hsa-mir-21-5p	1137.48 ± 311.15	896.77 ± 310.67	2255.3 ± 310.83	178.91 ± 311.1	1652.04 ± 349.34	1224.10	0.51
hsa-mir-320a	1098.31 ± 5.54	629.7 ± 5.53	1662.13 ± 7.4	230.57 ± 7.76	2044.63 ± 7.66	1133.07	0.67
hsa-mir-30a-5p	1019.32 ± 50.34	693.6 ± 50.38	1799.21 ± 50.27	244.18 ± 51.71	1746.44 ± 51.96	1100.55	0.77
hsa-mir-194-5p	937.8 ± 3.26	611.03 ± 3.27	1551.21 ± 3	21.82 ± 4.84	2085.16 ± 4.75	1041.40	0.5
hsa-mir-24-3p	1043.72 ± 14.88	596.78 ± 14.74	1446.12 ± 14.92	131.22 ± 14.81	1888.66 ± 16.75	1021.30	0.7
hsa-mir-29b-3p	1140.33 ± 239.43	548.05 ± 268.01	1377.94 ± 300.25	222.33 ± 326.85	1421.23 ± 343.78	941.97	0.48
hsa-mir-148a-3p	882.22 ± 44.32	685.86 ± 47.45	1573.63 ± 53.18	153.68 ± 61.77	1412.62 ± 68.81	941.60	0.55
hsa-mir-92a-3p	935.63 ± 394.22	669.9 ± 512	1972.25 ± 548.72	287.45 ± 625.47	517.12 ± 663.29	876.47	0.93
hsa-mir-130a-3p	858.18 ± 136.8	464.46 ± 136.32	1332.61 ± 136	184.57 ± 139.11	1335.17 ± 143.91	835.00	0.54
hsa-mir-192-5p	823.2 ± 27.03	463.05 ± 28.24	1199.48 ± 29.29	22.14 ± 28.64	1462.61 ± 27.3	794.10	0.45
hsa-mir-200a-3p	842.04 ± 216.67	461.93 ± 214.09	1213 ± 217.96	207.54 ± 239.75	1001.9 ± 259.84	745.28	0.71
hsa-mir-451a	585.15 ± 5.16	346.81 ± 5.12	1040.78 ± 5.1	18.21 ± 5.1	1144.71 ± 5.15	627.13	3.59
hsa-mir-221-3p	646.87 ± 254.66	331.6 ± 252.42	1168.93 ± 275.69	174.47 ± 306.5	702.78 ± 351.17	604.93	0.74
hsa-mir-17-5p	701.33 ± 116.93	329.25 ± 114.64	802.94 ± 125.29	189.49 ± 125.29	943.38 ± 147.03	593.28	14.83

hsa-mir-223-3p	491.82 ± 3.75	461.65 ± 3.72	766.15 ± 3.7	71.91 ± 3.68	1156.41 ± 3.67	589.59	4.21
hsa-mir-181a-5p	500.89 ± 3.18	258.43 ± 3.15	530.9 ± 3.12	73.23 ± 3.07	939.18 ± 4.31	460.53	0.8
hsa-mir-15a-5p	422.51 ± 36.88	244.35 ± 36.67	773.23 ± 41.82	45.83 ± 45.19	725.56 ± 46.37	442.29	0.55
hsa-mir-378a-3p	476.01 ± 80.34	289.39 ± 80.03	618.79 ± 79.7	26.96 ± 81.35	779.27 ± 82.07	438.08	0.48
hsa-let-7i-5p	445.56 ± 291.65	352.12 ± 292.48	779.72 ± 308.71	76.26 ± 313.46	526.95 ± 321.03	436.12	3.78
hsa-mir-200b-3p	435.09 ± 160.78	265.37 ± 159.48	637.7 ± 165.65	105.52 ± 166.51	669.94 ± 185.97	422.73	0.47
hsa-mir-19a-3p	268.54 ± 10.87	136.1 ± 10.86	409.82 ± 11.12	40.98 ± 11.63	501.02 ± 12.59	271.29	0.55
hsa-mir-375	154.44 ± 3.04	304.91 ± 2.89	459.13 ± 2.56	37.4 ± 2.53	334.31 ± 2.94	258.04	0.53
hsa-let-7g-5p	202.88 ± 268.06	136.15 ± 271.64	304.78 ± 271.06	95.73 ± 270.09	331.28 ± 268.54	214.16	0.75
hsa-mir-222-3p	213.44 ± 215.07	113.55 ± 211.03	324.65 ± 228.01	50.4 ± 294.26	247.69 ± 339.14	189.94	0.8
hsa-let-7d-5p	156.64 ± 4.87	79.11 ± 4.82	173.07 ± 4.83	61.09 ± 4.88	305.14 ± 4.82	155.01	0.6
hsa-mir-185-5p	162.83 ± 25.11	79.95 ± 29.6	207.83 ± 29.97	23.33 ± 30.59	290.11 ± 31.38	152.81	0.48
hsa-mir-34a-5p	138.73 ± 180.5	121.45 ± 178.06	279.19 ± 179.43	15.61 ± 189.58	181.21 ± 236.86	147.24	0.62
hsa-mir-146a-5p	114.99 ± 1.28	90.32 ± 1.28	208.5 ± 1.33	5.39 ± 1.33	295.21 ± 1.39	142.88	0.55
hsa-mir-26b-5p	132.52 ± 4.4	61 ± 4.35	173.8 ± 4.4	39.88 ± 4.64	254.22 ± 4.52	132.28	0.49
hsa-mir-187-3p	141.93 ± 274.84	54.98 ± 271.41	183.78 ± 311.43	53.44 ± 356.5	142.61 ± 413.21	115.35	1.25
hsa-mir-652-3p	100.17 ± 129.04	52.62 ± 127.3	160.76 ± 138.84	24.49 ± 139.11	146.2 ± 137.75	96.85	0.52
hsa-mir-181d-5p	72.57 ± 5.53	46.12 ± 5.51	120.99 ± 5.52	8.38 ± 5.53	143.73 ± 5.75	78.36	0.62
hsa-mir-708-5p	59.88 ± 6.66	33.13 ± 6.69	88.69 ± 6.71	2.87 ± 6.75	133.34 ± 6.78	63.58	0.66
hsa-mir-486-5p	69.19 ± 419.03	50.65 ± 418.55	134.69 ± 440.86	6.3 ± 471.08	51.35 ± 504.06	62.43	3.59
hsa-mir-146b-5p	30.86 ± 1.48	12.96 ± 1.46	48.91 ± 1.38	2.11 ± 1.37	97.28 ± 1.35	38.42	0.56
hsa-mir-503-5p	26.09 ± 0.43	19.98 ± 0.43	46.14 ± 0.69	3.31 ± 0.71	61.38 ± 0.9	31.38	0.43
hsa-mir-33a-5p	16.8 ± 68.03	6.02 ± 67.28	16.01 ± 67.56	3.71 ± 67.22	29.16 ± 71.33	14.34	0.62
hsa-mir-127-3p	18.23 ± 223.52	6.56 ± 232.17	20.4 ± 249.21	1.24 ± 269.65	20.84 ± 269.53	13.45	0.46
hsa-mir-214-3p	26.74 ± 281.19	4.52 ± 281.19	8.78 ± 286.08	0.57 ± 330.28	17.63 ± 326.45	11.65	0.77
hsa-mir-34c-5p	6.47 ± 0.06	11.47 ± 0.06	13.76 ± 0.1	0.34 ± 0.11	25.85 ± 0.11	11.58	0.6
hsa-mir-18a-5p	10.96 ± 36.06	7.54 ± 36.49	16.22 ± 41.71	3.9 ± 42.49	18.51 ± 41.61	11.43	3.07
hsa-mir-125b-5p	10.17 ± 3.14	7.57 ± 3.22	14.92 ± 2.82	1.24 ± 2.69	21.48 ± 2.72	11.08	0.89
hsa-mir-183-5p	10.37 ± 23.54	7.48 ± 23.32	12.1 ± 24.49	3.22 ± 23.96	18.77 ± 25.36	10.39	1.13
hsa-mir-96-5p	10.54 ± 173.75	9.67 ± 172.29	13.68 ± 177.22	1.62 ± 180.75	14.23 ± 185.21	9.95	0.5

hsa-mir-21-3p	11.5 ± 110.01	4.42 ± 109.93	10.09 ± 112.13	0.65 ± 125.48	17.27 ± 166.82	8.78	0.67
hsa-mir-122-5p	8.03 ± 215.18	10.04 ± 212.38	8.6 ± 253.73	5.7 ± 290.61	9.45 ± 282.3	8.36	2.57
hsa-mir-124-3p	3.23 ± 0.23	9.48 ± 0.23	12.86 ± 0.24	3.88 ± 0.25	10.84 ± 0.25	8.06	12.24
hsa-mir-877-5p	4.22 ± 0.05	5.96 ± 0.05	11.03 ± 0.06	1.47 ± 0.06	17.47 ± 0.08	8.03	1.21
hsa-mir-206	4.57 ± 0.08	8.26 ± 0.08	8.59 ± 0.15	0.61 ± 0.54	16.27 ± 0.64	7.66	7.01
hsa-mir-885-5p	5.96 ± 109.3	3.34 ± 110.35	10.59 ± 116.36	1.63 ± 115.64	8.95 ± 114.39	6.09	0.44
hsa-mir-193a-3p	5.65 ± 2.32	3.94 ± 2.41	6.79 ± 2.35	0.45 ± 2.64	13.24 ± 2.77	6.01	0.52
hsa-mir-296-5p	4.8 ± 1.81	3.91 ± 1.8	9.31 ± 2.14	1.28 ± 2.43	9.88 ± 2.71	5.84	3.88
hsa-mir-155-5p	4.26 ± 1.8	3.97 ± 1.76	3.54 ± 1.79	0.98 ± 1.86	9.92 ± 2.58	4.53	0.67
hsa-mir-101-3p	3.88 ± 160.58	1.68 ± 158.42	10.5 ± 204.41	0.33 ± 235.57	4.96 ± 259.02	4.27	0.42
hsa-mir-34b-5p	3.47 ± 1.3	4.08 ± 1.67	5.41 ± 1.77	0.58 ± 2.42	7.8 ± 2.4	4.27	0.39
hsa-mir-199a-3p	3.69 ± 31.61	1.97 ± 31.9	6.38 ± 32.15	0.76 ± 33.35	6.45 ± 37.6	3.85	1.07
hsa-mir-182-5p	3.27 ± 0.33	1.99 ± 0.43	2.43 ± 0.61	2.25 ± 0.69	8.86 ± 0.77	3.76	0.47
hsa-mir-410-3p	1.71 ± 1.04	0.8 ± 1.04	2.93 ± 1.13	0.14 ± 1.42	4.15 ± 2.01	1.95	0.45
hsa-mir-370-3p	1.07 ± 2.95	2.31 ± 2.93	1.61 ± 2.93	0.14 ± 2.91	2.54 ± 2.91	1.53	0.98
hsa-mir-571	0.69 ± 9.28	0.51 ± 9.24	0.76 ± 9.25	0.64 ± 9.24	1.33 ± 9.31	0.79	0.34
hsa-mir-199a-5p	0.53 ± 293.11	0.31 ± 298.86	1.39 ± 311.93	0.21 ± 314.11	0.63 ± 350.34	0.61	0.54
hsa-mir-337-3p	0.28 ± 2.58	0.26 ± 2.58	0.26 ± 2.58	0.21 ± 2.73	0.53 ± 3.32	0.31	0.59
hsa-mir-183-3p	0.28 ± 3.96	0.16 ± 5.79	0.31 ± 6.09	0.13 ± 5.96	0.52 ± 5.95	0.28	0.8

Table S2. Association between the selected miRNAs and cancer were found using the miR2 disease database (http://www.mir2disease.org/), and some selected references retrieved by the database are displayed below.

**miRNA**	**Cancer type**	**Reference**
hsa-let-7d-5p	Prostate	Porkka, K.P.; Pfeiffer, M.J.; Waltering, K.K.; Vessella, R.L.; Tammela, T.L.; Visakorpi, T. MicroRNA expression profiling in prostate cancer. *Cancer Res.* **2007**, *67*, 6130–6135. PMID:17616669.
hsa-let-7i	Ovarian	Yang, N.; Kaur, S.; Volinia, S.; Greshock, J.; Lassus, H.; Hasegawa, K.; Liang, S.; Leminen, A.; Deng, S.; Smith, L.; et al. MicroRNA microarray identifies Let-7i as a novel biomarker and therapeutic target in human epithelial ovarian cancer. *Cancer Res.* **2008**, *68*, 10307–10314. PMID:19074899.
hsa-miR-21	Prostate	Prueitt, R.L.; Yi, M.; Hudson, R.S.; Wallace, T.A.; Howe, T.M.; Yfantis, H.G.; Lee, D.H.; Stephens, R.M.; Liu, C.G.; Calin, G.A.; et al. Expression of microRNAs and protein-coding genes associated with perineural invasion in prostate cancer. *Prostate* **2008**, *68*, 1152–1164. PMID:18459106.
hsa-miR-21	Bladder	Dyrskjt, L.; Ostenfeld, M.S.; Bramsen, J.B. Genomic profiling of microRNAs in bladder cancer: miR-129 is associated with poor outcome and promotes cell death in vitro. *Cancer Res.* **2009**, *69*, 4851–4160. PMID:19487295.
hsa-miR-22	Prostate	Porkka, K.P.; Pfeiffer, M.J.; Waltering, K.K.; Vessella, R.L.; Tammela, T.L.; Visakorpi, T. MicroRNA expression profiling in prostate cancer. *Cancer Res.* **2007**, *67*, 6130–6135. PMID:17616669.
hsa-miR-30c	Prostate	Volinia, S.; Calin, G.A.; Liu, C.G.; Ambs, S.; Cimmino, A.; Petrocca, F.; Visone, R.; Iorio, M.; Roldo, C.; Ferracin, M.; et al. A microRNA expression signature of human solid tumors defines cancer gene targets. *Proc. Natl. Acad. Sci. USA* 2006, 103, 2257–2261. Epub 2006 Feb 3. PMID:16461460.
hsa-miR-30c	Bladder	Wang, G.; Zhang, H.; He, H.; Tong, W.; Wang, B.; Liao, G.; Chen, Z.; Du, C. Up-regulation of microRNA in bladder tumor tissue is not common. *Int. Urol. Nephrol.* **2009**. [Epub ahead of print]. PMID:19475496.
hsa-miR-92a	Hepatocellular	Connolly, E.; Melegari, M.; Landgraf, P.; Tchaikovskaya, T.; Tennant, B.C.; Slagle, B.L.; Rogler, L.E.; Zavolan, M.; Tuschl, T.; Rogler, C.E. Elevated expression of the miR-17-92 polycistron and miR-21 in hepadnavirus-associated hepatocellular carcinoma contributes to the malignant phenotype. *Am. J. Pathol.* **2008**, *173*, 856–864. Epub 2008 Aug 7. PMID:18688024.
hsa-miR-192	colorectal	Braun, C.J.; Zhang, X.; Savelyeva, I.; Wolff, S.; Moll, U.M.; Schepeler, T.; Ørntoft, T.F.; Andersen, C.L.; Dobbelstein, M. 53-Responsive micrornas 192 and 215 are capable of inducing cell cycle arrest. *Cancer Res.* 2008, 68, 10094–10104. PMID:19074875.
hsa-miR-451	colorectal	Bandres, E.; Bitarte, N.; Arias, F.; Agorreta, J. microRNA-451 regulates macrophage migration inhibitory factor production and proliferation of gastrointestinal cancer cells. *Clin. Cancer Res.* 2009, 15, 2281–2290. Epub 2009 Mar 24. PMID:19318487.

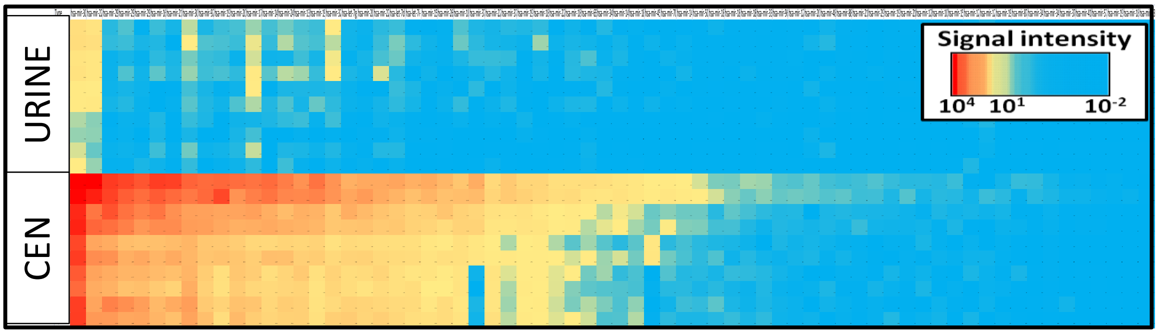

Figure S1. Heatmap of signal intensity for Multiplex miRNA assay comparing extracellular vesicles (EVs) isolated from 10 mL of urine using CEN method, with urine (150 µL).

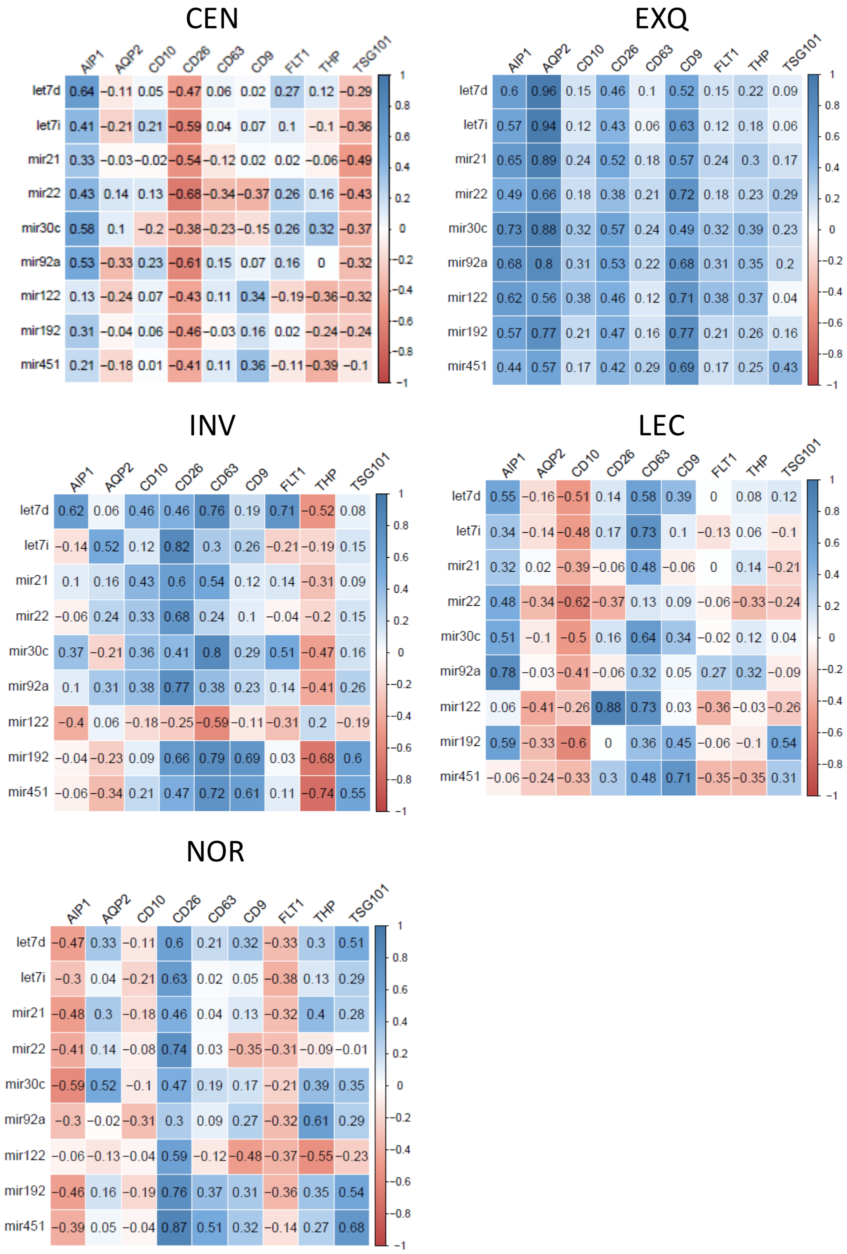

Figure S2. Matrix correlations between miRNAs content observed by the multiplex miRNA assay (expressed as signal intensity) and protein content, expressed as densitometry values from Western-blotting for different proteins (data obtained from Royo et al. [13]). Values are correlation coefficients.

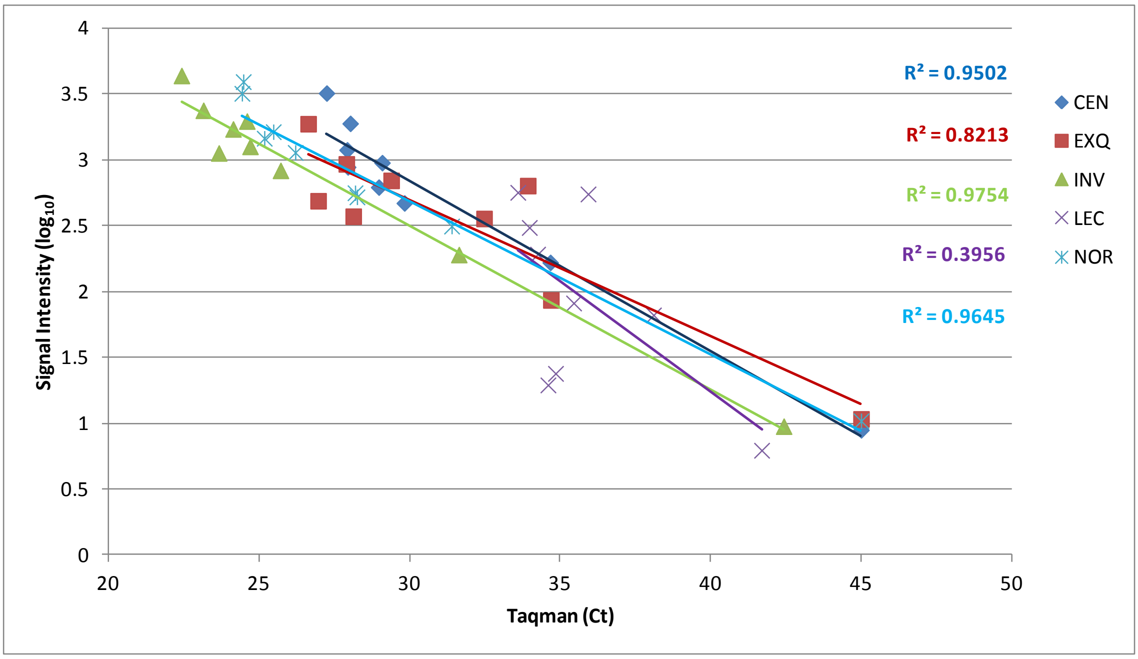

Figure S3. Correlation analysis between Taqman quantitative PCR (qPCR) and signal intensity from the Multiplex miRNA assay. Each method is treated independently, thus each single point corresponds to the measure of a unique miRNA obtained by a unique method, averaged for all the samples.

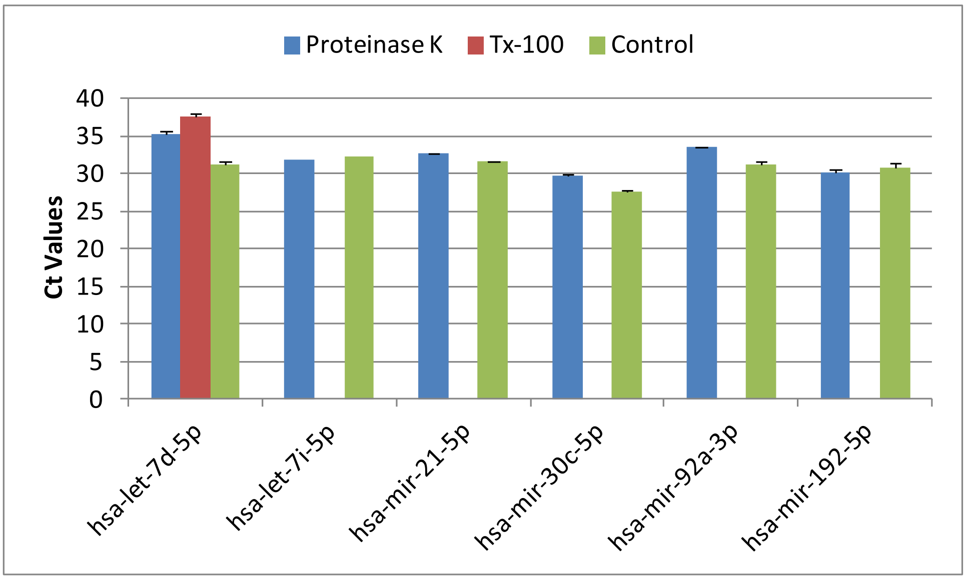

Figure S4. RNAse protection assay. Urine samples were treated with proteinase K or Triton X-100 before incubating with RNase, and uEVs were obtained using NORGEN reagent before proceeding with miRNA qPCR amplification. Note: miRNA amplification was mostly resistant to proteinase-K+RNAse treatment, and only when using RNAse in the presence of 0.1% Triton X-100 was the amplification abrogated or reduced (hsa-let-7d-5p). Error bars are SD of technical replicates (n = 3).

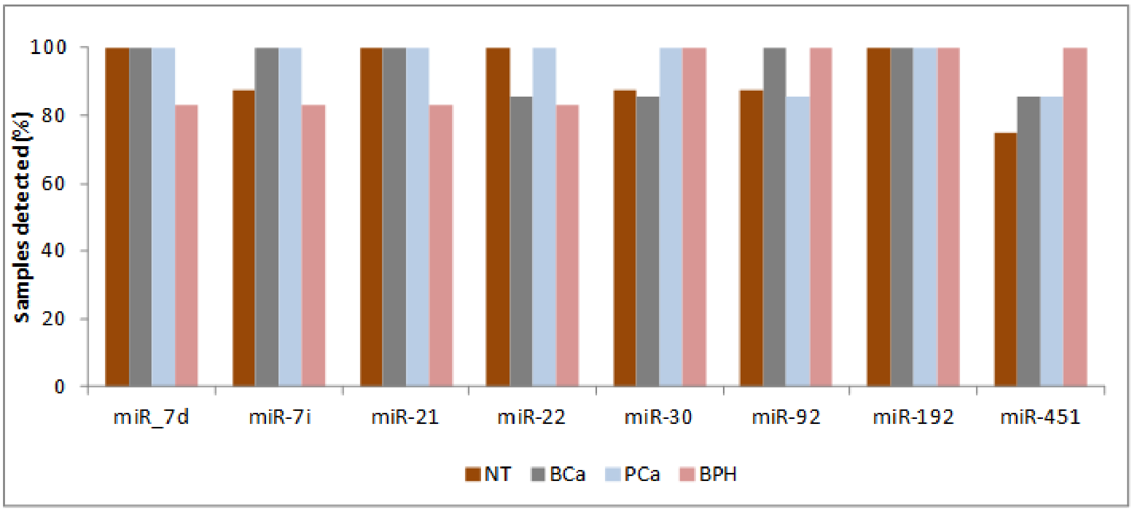

Figure S5. Percentage of detection for each group of patients and miRNA. NT: non-tumor; BCa: bladder cancer; PCa: prostate cancer; BPH: benign prostate hyperplasia.
